# Smart Mask as Wearable for Post-Pandemic Personal Healthcare

**DOI:** 10.3390/bios13020205

**Published:** 2023-01-30

**Authors:** Jingcheng Li, Jing Yin, Seeram Ramakrishna, Dongxiao Ji

**Affiliations:** 1 Centre for Nanotechnology and Sustainability, Department of Mechanical Engineering, National University of Singapore, Singapore 117081, Singapore; 2 National Engineering Laboratory for Modern Silk, College of Textile and Clothing Engineering, Soochow University, Suzhou 215021, China; 3 College of Textiles, Donghua University, Shanghai 201620, China

**Keywords:** smart wearables, respirator, sensor, intelligent materials, sustainability

## Abstract

A mask serves as a simple external barrier that protects humans from infectious particles from poor air conditions in the surrounding environment. As an important personal protective equipment (PPE) to protect our respiratory system, masks are able not only to filter pathogens and dust particles but also to sense, reflect or even respond to environmental conditions. This smartness is of particular interest among academia and industries due to its potential in disease detection, health monitoring and caring aspects. In this review, we provide an overlook of the current air filtration strategies used in masks, from structural designs to integrated functional modules that empower the mask’s ability to sense and transfer physiological or environmental information to become smart. Specifically, we discussed recent developments in masks designed to detect macroscopic physiological signals from the wearer and mask-based disease diagnoses, such as COVID-19. Further, we propose the concept of next-generation smart masks and the requirements from material selection and function design perspectives that enable masks to interact and play crucial roles in health-caring wearables.

## 1. Introduction

Air is one of the fundamental survival needs of most creatures. Air quality as a key assessment of life standards, has a huge impact on human health. Recovering and thriving from the global pandemic [1], the rising public awareness of respiratory system conditions witnessed boosted interest in commerce and research in related fields [2,3,4]. When covering the nose and mouth of the wearer, a face mask should filter out respiratory pathogens and particulate matter to prevent these harms from getting into our respiratory systems during inhaling, which is the primary route of COVID-19 transmission [4].

Mask can be a necessary, reasonable and effective non-pharmaceutical intervention against rival agents that travel and are carried by aerosol and droplets, in many cases, to reduce the transmission efficiency of virus and its secondary transmission [5,6,7]. Taking the fight against COVID-19 as an example, Leung and coworkers reported that the decrease in virus transmission rate is shown to be nearly linear, proportional to the product of mask effectiveness and coverage rate [6]. This means that with timely and comprehensive application of masks, a secondary wave of COVID-19 can be effectively prevented together with other non-pharmaceutical measures. Notably, they proved that even relatively ineffective face masks, if adopted broadly, still shield virus in community transmission, alleviate hospitalization and decrease deaths. The use of face masks helps the general public by providing both prevention of illness for healthy ones and transmission of asymptomatic [8]. Larger droplets settle down easily due to gravity, while respiratory droplets and aerosols with a smaller size (<5 μm) carry and help viruses such as SARS-CoV-2 transmit [9]. These airborne transmissions through aerosols and droplets efficiently fasten the virus spread among human and/or animals, and eventually provoke pandemic outbreaks.

On the other hand, as an effective personal protection procedure, wearing face masks helps protect both the wearer and the others who surround them. In this case, a mask as a filtering device also functions as a “source control” [4,10], preventing the wearer’s respiratory droplets and particles from traveling into the air and onto other susceptible people or objects during coughing, sneezing or talking, especially when the wearer is suffering from respiratory diseases.

**Figure 1:**
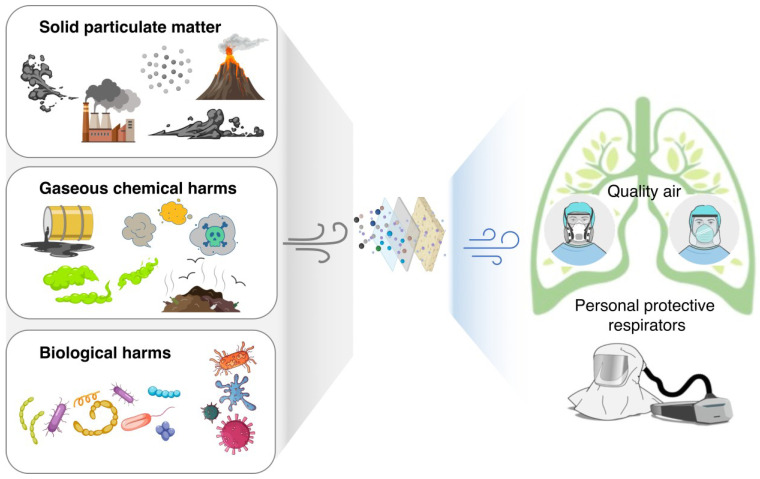
Mask filters out pathogens from the environment and provides wearers with quality air.

The necessity of wearing face masks lies not only in fighting pandemics such as COVID-19. Apart from providing protection against the spread of respiratory disease, in the post-pandemic state, masks serve as an effective PPE in workplaces, as some hazards might remain despite engineering controls and safe systems of work are in place. For example, depending on filtration efficiency defined from various national and international standards, masks can filter out particles with a size as small as 10–500 nm [11], and mitigate odor, smell or toxic gases to some extent if equipped with absorbing medium to convert them into less harmful forms (Figure 1). Specifically, solid particulate matter that can be trapped and held in a mask filter includes large particles such as grain or/and pollen (15 μm), respiratory droplets (5–10 μm), to smaller ones that are harder to trap, such as dust particle (1–10 μm), fume and smoke (0.4–0.7 μm). Mask helps to filter out these particulate matters that may cause allergic reactions or even biological harm—microorganisms like viruses and bacteria (0.1–3 μm) that could lead to infection [12,13,14]. Additionally, filters have been designed to protect against both solid particles and liquid particles (mists, fine sprays and aerosols). For instance, masks equipped with gas absorption respiratory cartridge filters mitigate the harm from gaseous chemical harm, which includes Volatile Organic Compound (VOC) that evaporate easily and can produce strong odors, and inorganic gaseous pollutants that have different effects on human health and the environment, such as carbon monoxide (CO) and nitrogen oxides (NO_x_), sulfur dioxide (SO_2_), ammonia (NH_3_), chlorine (Cl_2_), formaldehyde (HCHO) and Radon (Rn) [15]. VOCs can have both short-term and long-term health effects, such as headaches, eye and respiratory irritation, and even cancer. It is important to take proper precautions when working with or around products that contain them, such as paints, cleaning supplies, building materials, and sources like vehicle exhaust and industrial emissions [16].

Thus, defending with personal protective equipment where other methods are not effective or inadequate is the last vital resort to protection, and the need for a filtering medium to minimize the impact of these hazards becomes crucial to human health [17,18,19]. With growing concerns about reducing and eliminating the harmful impacts of these risks, air filtering masks have sought out to be one of the solutions that provide protection to lungs and other organs of the human body from air pollution. Although nose and throat can trap larger particles typically above 10 microns and lungs are able to clear themselves, long-term exposure to unhealthy air conditions and excessive inhalation of dust introduces acute and chronic harm to the respiratory system and organs, which leads to boosted possibilities of respiratory irritation and infections, asthmatic attacks, and vascular diseases [12,20]. In this regard, engineering control methods should be introduced if substitution of hazardous substances with non-hazardous substances is not possible [21], and masks should be adopted as suitable personal protective equipment.

According to the World Health Organization (WHO), a surgical mask, able to block particle sizes larger than 5 μm with at least 90% efficiency, is not classified as respiratory protective equipment that uses filters to remove contaminants from the air being breathed in [21]. However, in this review, the term “mask” is used with a broader aspect that refers to a device that covers the mouth and nose area of the wearer to provide filtering and other functionalities subject to demands, as discussed in the following sections. Several works have focused on comparing the effectiveness of filtration performances of masks (mainly N95) or surgical masks with distinct function/material/structure or providing comparisons on market-available commercial products. In this review, we first present the current filtration strategies of a mask, including filtration mechanisms and how its filtration performance and functionalities rely on its structural designs. Among various manufacturing approaches to respiratory filters, we justify the advantages of the electrospinning process and then discuss the material selection, including the base substrate material and the functional additives that introduce designed properties to the fibers. Meanwhile, we emphasize the transition and correlation between conventional commonly used masks, which serve solely as filters, and recently developed smart masks. We emphasize that with intelligent and green materials, the integration of various functional modules provides traditional filtering masks with the capability to sense and provide physiological information to the wearer that can be helpful for health monitoring or disease diagnosis and moving further, the potential of wearable applications.

## 2. Filtration Strategies in Masks

Filtration of gas refers to the physical process by which a porous filtering medium, in many cases fibrous material, traps and eliminates particles when a gas stream passes through it, thus enhancing the quality or purity of the filtered product [22,23,24]. The fundamental purpose of using a mask is to filter out particles in the air that can be harmful to our health. Currently available masks, either as research prototypes or commercial products in the market, have been adopting diverse strategies but share the same goal of making the filtration media work with a higher efficiency and lower pressure drop.

### 2.1. Structural Design and Filtration Mechanism

A layer-structured mask is generally composed of several layers, each serving an individual purpose: an essential filter layer to capture and block particles, a hydrophobic layer to protect the mask from possible exposure to droplets and liquids, and a supporting layer to hold up the whole mask and comfortably contact the wearer’s skin. The filtration efficiency and other additive functionalities of masks are largely dependent on the structure of the filtering media, in most cases, nanofiber membranes. Among various types of mask filtration media, fiber-based filters, porous activated carbon, or porous ceramics, filters with a fiber base have been found to be used widely due to their edges in versatility of production, adjustable structure combination and morphology [25].

**Figure 2:**
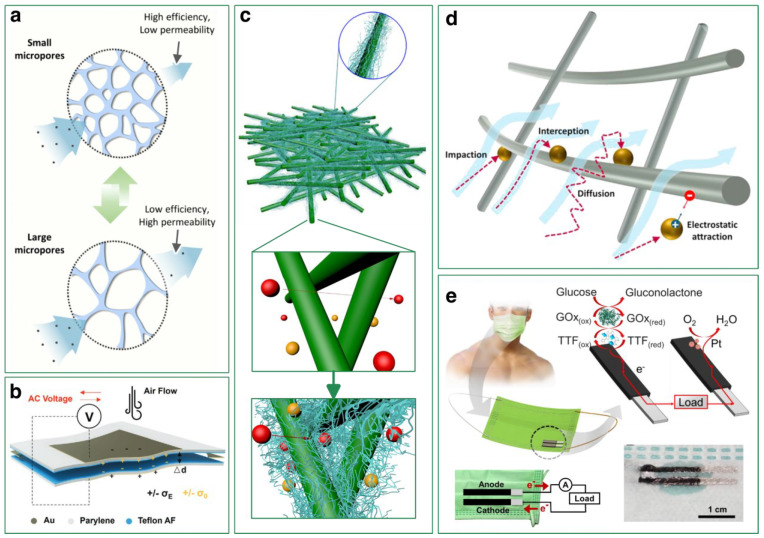
Filtration performance and functionalities of the mask rely on its structural designs: (**a**) Dynamic adjustment of micropore sizes of an elastic nanofiber-based air filter (Adapted and reproduced with permission from Ref. [29]. Copyright 2021, American Chemical Society); (**b**) A self-powered pressure sensor on mask for breath detection enabled by electrostatic induction effect (Reproduced with permission from Ref. [30]. Copyright 2022, Wiley-VCH GmbH, Weinheim); (**c**) Fibrous membrane for air filtration with a hierarchical structure (Adapted and reproduced with permission from Ref. [31]. Copyright 2018, Elsevier B.V.); (**d**) Particulate matter capture mechanisms: electrostatic attraction, diffusion, interception and impaction (Adapted and reproduced with permission from Ref. [23]. Copyright 2021, Elsevier B.V.); (**e**) Printed electrodes on mask as biosensor that detects glucose level continuously (Adapted and reproduced with permission from Ref. [32]. Copyright 2022, Elsevier B.V.).

A properly designed structure supports and realizes the optimization of a mask’s performance, including air filtration. For example, a nanofibrous membrane prepared by electrospinning that possesses a bead-on-string structure significantly improves its air filtration performance, as shown by its higher filtration efficiency and lower pressure drop compared with a smooth structure [26,27,28]. This is because nanofibers with such a unique structure have an increased inter-fiber distance, hence resulting in a decreased volume fraction, making it easier for air to flow through. Figure 2a shows an electrospun nanofiber-based filter that modulates and rescales its pore size dynamically according to the air quality of the ambient environment and the wearer’s breathing demand, hence offering adaptive respiratory protection to the wearer [29]. As Jaeho et al. reported, this dynamic micropore rescaling is achieved by strain control of the elasticity of a stretchable fiber membrane, with homogeneous expansion and shrinkage of the elastic membrane made with poly(styrene-b-butadiene-bstyrene) when a change in mechanical strain is applied. This response is practice, direct, possible to quantify or simulate and implementable compared to that responds to chemical or thermal stimuli.

On the other hand, nanofibers are used not only for its functionality of filtration, but also to fulfil the demands of achieving other purposes and realizing combined functionalities for the mask due to their unique structure, such as self-powered sensors when introduced with PE/TE mechanisms [33,34,35]. This generally comes from the inherent property of specific polymer material used for nanofiber production, in that the variation in polarity and arrangement of the polymer molecular chain leads to divergent abilities of piezoelectric responses when applying pressure, or triboelectric effects when periodically contact and separate with another material properly selected with divergent affinity to get or lose. Zhong et al. developed an ultrathin self-powered pressure-responsive face mask taking advantage of electrostatic effects and high electrostatic induction efficiency of piezoelectret parylene/Teflon AF films [30] as shown in Figure 2b. The mechanical–electrical signal conversion takes place when airflow is introduced to change the distance between two Teflon AF films. This change in air gap thickness Δd consequently decides the charges that parylene films hold transported by Au electrodes, during which the voltage was generated.

Another aspect to consider during structure design is hierarchy. By creating a nanostructure, it is possible to control the fundamental properties of materials without changing their chemical composition. In other words, the multi-level structural design of nanofibers can lead to specific structures that hold unique functionality that greatly improves filtering performance greatly [25,36,37], which is exemplified in the designs of nanofiber with honeycomb [38], beads-on-string [39], nano nets [40], or lotus leaf inspired structures [41] fabricated by electrospinning. For example, Figure 2c depicts a schematic of hierarchically grown ultra-fine SiO_2_ nanofilaments on a scaffold poly(m-phenylene isophthalamide) (PMIA) network with zooming in scale levels, as shown downwards on the picture [31]. This hybrid double network membrane efficiently filters out PM2.5 and PM10 particles, with significant improvement compared with a bare PMIA membrane that does not have a hierarchical structure, of which the removal efficiency reached 97.33% and 98.48%, respectively. In addition, the porosity and specific surface area are greatly promoted with the morphology change, whereas the mechanical strength of PMIA nanofibers is preserved. Another case of hierarchical design in filtration membranes, for example, is a silver nanowire percolation network for particulate matter air filtration developed by Jeong and co-workers [42]. In this work, Ag nanowires were evenly distributed on a nylon mesh, which serves as a backbone supporter, through vacuum filtration to create a metal nanowire network that captures PM2.5 with an efficiency as high as 99.99%. Nylon fiber covered with a uniform network of metal nanowires creates a spider web-like structure; the welded junctions between Ag nanowires promote its electrical connection and mechanical properties as well. Notably, distinguished from commonly used polar polymer nanofiber filters that depend on the physical filtration process, this air filter can remove PM2.5 particles actively by controlling the applied voltage to vary the applied electrostatic force and Ag nanowires as the transparent conducting electrode.

PM particles can be captured mechanically by the filter through impaction, interception, diffusion or electrostatic attraction, illustrated in Figure 2d. It is clear that particles with diameters bigger than the filter’s pore size will be stopped and trapped. For particles that possess a smaller size, different capturing mechanisms apply [23]. For airstream passing through the filter too rapidly for the carried particles to adjust their streamlines, particles will be trapped by inertial impaction and prevented from continuing their routes. This mechanism relies on inertia and usually occurs for comparatively larger particles [24]. Interception happens when a particle carried by a streamline that possesses a radii bigger than the distance from the fiber to the particle. Provided not enough distance, the particle is captured by the fiber, especially when the ratio of the particle and fiber diameters (the interception parameter $d_{p}/d_{f}$) falls in specific regions. This mechanism occurs for most submicron-sized particles [43]. Filtration via diffusion works because of particles’ Brownian motion, which allows particles to escape from their streamlines randomly impacted by filtration fibers with kinetic energy. This mechanism even applies to smaller particles, which are difficult to capture under other mechanisms due to their tiny size and negligible weight [23]. Particles and fibers with unipolar or bipolar charges, or either of them in a neutral state and polarized by charging the other, attract each other due to Coulombic forces in the former case or dielectrophoretic forces in the latter. This is how filtration via electrostatic attraction works [44,45,46]. It is worth noting that under this mechanism, the airstream is not affected; that is to say, filtration efficiency can be enhanced without the sacrifice of permeability. However, it remains an issue to quantify precise charge distributions on the contacting surfaces, which makes particle-capturing behavior unpredictable [24].

Figure 2e shows a research prototype of the integration of a glucose biosensor on a mask via electrode printing developed by Itthipon and co-workers [32]. They screen-printed CNT-based ink on the medical mask fabric as electrodes, and electrodeposited polyaniline (PANI) together with CNTs as anodes and poly(3,4-ethylenedioxythiophene) (PEDOT) as oxygen-reduction-based cathodes. The bioanode was modified by using oxidase enzyme and electrochemical mediator to provide bio-electrocatalytic conversion of glucose with lower potential. Additionally, a catalytic layer of biocompatible chitosan polymer was coated onto the electrode for protection, preventing the leakage of active components. As the wearer’s sweat drops on the anode, the glucose contained in the sweat is enzymatically oxidized, and the released electrons go through the conductive electrodes and thus generate current responses. In this way, the biocatalytic system detects glucose, and the responsive current output is shown to be proportional to the glucose concentration, with a detection range of 0.22–10 mM. This work implies the applicability that a mask-based, self-powered biosensor is able to work for digital healthcare diagnosis and disease monitoring, regardless of the disturbances from lactic acid, uric acid, ascorbic acid or creatinine.

On a macroscope, the configuration design of the mask involves proper ergonomics so that the facial area mask covers performs a 3D shape rather than a flat surface. In other words, the filtration efficiency of a certain material tested under the condition that near-zero leakage is assumed may not represent what happens in the real-case scenarios, where leakage along edges of the mask can be expected, especially when expiratory events with high pressure such as cough occurs [47,48]. This requires the mask to preserve its filtration efficiency on a curvilinear 3D freeform surface, where fabrication methods for functional circuits can be helpful [49].

### 2.2. Material Selection

**Figure 3:**
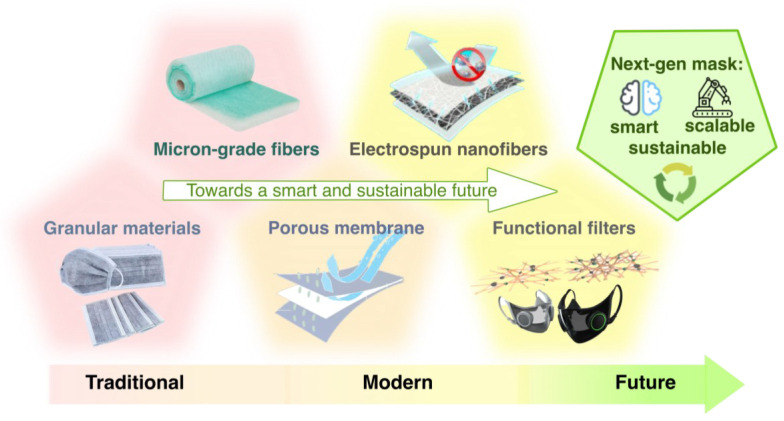
Mask filtration material development: a trend to higher porosity and improved filtration efficiency.

Diverse materials have been adopted as filter media, subject to specific needs and requirements. Generally, traditional air filters involve air filters made by porous membrane or micron-grade filtration medium, such as ultra-fine glass fiber and melt-blown electret micron-fiber, while increasing recent focus on electrospun nanofiber and other functional filtering materials contributes to the development of modern filter materials with better filtration performances thanks to their higher porosity and thinner fiber diameter (Figure 3). Millimeter- and micron-level particulate filtration materials, such as charcoal and granular activated carbons, are widely used traditional filtration media due to their heat resistance, chemical resistance, and price advantages. These advantages help them become the mainstream filtration material for mask filters. However, as far as they serve well on some occasions, the issue exists that their filtration efficiency decreases rapidly as filtered particles accumulate and block the pores, thereby increasing air resistance and making masks made by these materials unsuitable to wear [17,50,51,52].

In addition, material selection of the masks ought to hold the consideration that masks are continuously immersed in the environment near our respiratory system, where a general high humidity can be expected. This indicates that a hydrophobic or water-repellent surface is preferable because they can help to prevent droplets of moisture such as those from breath or saliva from permeating the mask, and consequently, help to prevent the mask from becoming wet, which may jeopardize the effectiveness of mask filtering out particles and make it more uncomfortable to wear. Additionally, a hydrophobic surface can help prevent the filtration efficiency drop over time by preventing the build-up of bacteria and other microorganisms on the mask [48,53,54]. Therefore, hydrophobic modification should be considered for anti-humidity performance. However, researchers have also found that hydrophilic properties can be desirable occasionally. For instance, for cloth masks made with inherently hydrophilic fabrics, a high humidity condition mimicking respiration leads to an increased filtration efficiency of hygroscopic nanoparticles, such as respiratory droplets [53]. This will benefit the pathogen propagation reduction because humid supplied by exhalation contributes to swelling of the hydrophilic fibers, including H_2_O adsorption onto its surface and uptake within the fibers.

#### 2.2.1. Nanofiber-Based Filters

Conventional filtration materials have certain limitations when it comes to fine particle excerpts, especially with particulate materials of less than 2.5 nm [55,56]. On the other hand, filters with nanofibrous materials have shown a higher capacity to hold contaminate, a prolonged work life, and improved filtration efficiency due to their high porosity, favorable nanoscale inter-connectivity, and fine pore size [57]. Thus, nanofiber-based filtration media in different forms, such as nanoparticle-doped fibers, sing-polymer-based fibers, or multi-polymer composites, have garnered significant popularity [58], and made them an excellent choice for base material in PPE, such as respiratory protection against high dust concentration in coal mining workplaces [25]. Among nanofiber-based filters, nanofibers prepared by the electrospinning approach are of the most interest. Electrospun nanofibers block fine particles due to their high specific areas and porous structure with tunable diameters [59].

Innovative designs of air filters consider filtration compounds to play an essential role. For example, poly(vinylidene fluoride) (PVDF) is a commonly used membrane material for filtration with outstanding thermal stability, dielectric property and chemical resistance [60,61,62]. Ding et al. reported an electret air filter media [63] taking advantage of the high dipole moment of PVDF, which interacts with modified SiO_2_ nanoparticle which enhances the interfacial charge by grafting reaction. Nanofibers based on other polymers as filtration media have been reported, such as polyamide 6/6 (PA 6/6) [64], PMIA [65], polyvinyl alcohol (PVA) [66], polyacrylonitrile (PAN) [36] etc. In addition, nanofiber-based membranes can hold and carry additives, such as nanoparticles or nanofillers, with specific functionalities, which opens broad opportunities for nanofibers in filtration applications.

#### 2.2.2. Functional Additives

Functionalities can be achieved intrinsically or introduced extrinsically, as is fulfilled by the filtering material itself or, more commonly, the latter case, by integrating electric components on the commercially available surgical masks. Various additives with unique inherent characteristics have been developed to introduce new functionalities or properties for enhanced nanofibers. Table 1 shows typical additives in nanofibers that introduce functionalities to the filter or enhance filtration performance. For instance, silver particles or silver nanowires are often used for antibacterial purposes, incorporation of iron oxide nanoparticles often contributes for magneto responsive properties [42,67,68,69], or introducing hydrophilic or negatively charged surface modifications for the antifouling ability of filtration membranes [52,70]. In addition, various activation active reagents or dopants of functional groups provide another prospect for defining new surface properties of nanofiber membranes to qualify gas filtration, such as toxic gas adsorption [56]. In the case of developing virus or anti-microbial air filters, these agents can be polypeptides or metals, such as copper or silver, or organic acids. Some inherently anti-bacterial polymers, such as chitosan, with its deacetylation degree and free amino group that chelates and is charged positively to attract negatively charged bacterial surfaces, are applicable to processes directly as well [71]. As such, filters with these functional additives are capable of filtering out airborne transmission of viruses, fungi or bacteria carried by particulate matters [72], or deposit/coat anti-bacterial agents directly onto the membrane post-electrospinning [73].

It is worth noting that the hierarchy of nanoparticle size of additive matters. As Cao and coworkers reported, TiO_2_ nanoparticle size has a great impact on the structure and performance of PVDF membrane [60]. Their permeability test results confirm that TiO_2_/PVDF membranes with smaller nanoparticles have smaller mean pore sizes on their surfaces and more apertures inside the membrane, which also improves their antifouling ability. The charge storing ability of TiO_2_, as well as Si_3_N_4_, SiO_2_, or boehmite, as Table 1 shows, makes them candidates for introducing quasi-static fields in air filtration membranes [56,74]. For respiratory air filtration that tries to minimize the risk of pathogen-caused infection, anti-bacterial and anti-viral properties are often desired since viruses and bacteria are always carried by dust particles [25,75]. This can be achieved by introducing nanosized additives of silver or other metals. What’s more, inorganic substances and metal additives that serve corresponding purposes make functionalized nanofibers possible. Recently, metal–organic frameworks (MOFs) have been heatedly investigated in toxic gases and vapors capturing, sensing and removing by catalytic degradation due to their controllable size and molecular-accessible pore walls [76]. For example, Cu_3_(BTC)_2_ improves ammonia adsorption because of the presence of Cu^2+^ open metal cites, which behave as Lewis acids to coordinate NH_3_ Lewis bases and react with ammonia to form a presumed diammine–copper(II) complex, making it irreplaceable by porous substitutes such as activated carbon [76,77,78]. Apart from nanoparticles, nanowires with desirable size scale and aspect ratio are qualified to fulfil specific mechanical, thermal or electrical properties as well [79]. For instance, ultralong hydroxyapatite (HAP) nanowires and cotton (CT) fibers are intertwined to form air filter paper [80], where the application of HAP nanowires successfully avoids the disadvantages of its counterparts, possesses neither the biological toxicity of carbon nanotubes, nor the environmental friendliness of polymer-based nanofibers.

Apart from introducing additives to nanofiber-based mask filters during the manufacturing process, efforts have been made to functionalize commercially available encapsulated masks, for instance, through laser-induced graphene [81,82]. Li’s group reported a laser-induced graphene-based self-cleaning mask. By introducing a graphene layer, the polyimide surface of the treated surgical masks becomes superhydrophobic and thus bounces off the droplets, contributing to its self-cleaning characteristic combined with photothermal-enabled sunlight sterilization [83]. A similar approach was adopted and improved by monitoring mask conditions via moisture-induced electricity of gradient graphene, as proposed by Huang and coworkers [84].

**Table 1:** Typical additives in nanofibers that introduce functionalities to the filter or enhance filtration performance.

Base Fiber	Additive(s)	Introduced Properties and Advantages	Ref.
Cellulose	MTMS ^1^	Silanization modification to obtain super-hydrophobicity	[85]
Cotton	Hydroxyapatite nanowire	Biocompatibility, environmental friendliness, improved flexibility	[80]
Nylon	Ag nanowire	Transparent conductor for electrostatic field with excellent conductivity and electrical stability	[42]
PAN	Ag nanowires/NP ^2^	Antibacterial, antiviral	[68,86]
PMIA	LiCl	High conductivity to endow Taylor cone during electrospinning	[65]
PPy ^3^	Nanoscale Cu_3_(BTC)_2_(H_2_O)_3_ MOFs ^4^	Gas capture and gas detection of ammonia	[78]
PU ^5^	LiCl	Reduce membrane pore size, robustize mechanical property, enhance purification capacity	[87]
PU	Si_3_N_4_, SiO_2_	Serve as ferroelectric inorganic electrets during fabrication, improves mechanical property	[74]
PVA, PEO ^6^	Nitrogen-doped TiO_2_	Makes fiber photocatalytic, bacteria disinfection under light irradiation	[88]
PVDF	TiO_2_ NP	Makes fiber photocatalytic, antifouling, helps crystallization of PVDF molecules	[60]
PVDF	GPS ^7^ modified SiO_2_ NP	Charge enhancer that improves electret effect	[63]
PVDF	ZnO nanowires	Introduced semi-conductivity for photodetectors, enable toxic gas detection	[89]

^1^ MTMS: methyltrimethoxysilane. ^2^ NP: nanoparticle. ^3^ PPy: polypyrrole. ^4^ BTC: benzene-1,3,5-tricarboxylate. ^5^ PU: Polyurethane. ^6^ PEO: poly(ethylene oxide). ^7^ GPS: γ-glycidoxypropyl trimethoxysilane.

### 2.3. Electrospinning as an Approach for Filter Membrane

Manufacturing methods of mask filter membranes vary, where the functional filtration layer of the most commonly used surgical masks is usually composed of nonwoven fabrics, which provide better filtration performance than woven and knit fabrics because nonwoven fabric-based filters have thicker 3D structures and increased distance to stop the passing particles [2,86]. Among the three main nonwoven fiber manufacturing methods, as in melt-blowing, electrospinning and spun-bonding, fibers produced by the first two approaches possess finer pore size and smaller fiber diameter. Comparative evaluations of nanofiber-based filter and melt-blown filter have shown that the former showed excellent reusability as mask over melt-blown filter and that it exhibited consistent high performance in terms of filtration efficiency even after 10 spraying cleaning cycles when preserving its higher water vapor transmission permeability and good hydrophobicity [90], making it a suitable choice for wide use in mask applications.

Therefore, electrospinning, as the most common approach to nanofiber production, should be emphasized. Electrospinning provides feasibility and realization for the design and formation of nanofiber membranes with versatile yet unique structures and morphologies [86,91,92,93]. Generated nanofibers with semi-ordered or disordered systems are of great interest for their applications in air filtration. With diameters of the electrospun fibers reduced to nanometre scale, one could observe desired properties such as an increase in surface to volume ratio and a strengthen in mechanical behavior arise [94]. Nanofibers prepared by electrospinning have many outstanding features, including a large surface area to volume ratio, adjustable fiber diameters and pore sizes that can be controlled by altering working parameters during electrospinning [62], simplicity in preparation and fabrication setup, etc. that lead to higher PM filtration efficiency [95]. Research and interests have been shown and investigated in adopting functions to tune air filtration performance, as mentioned in the discussion of mask filtration media structural design. Superhydrophobic surfaces achieved by bead-on-string fiber morphology are a good illustration [28]. To add on, electrospinning is a low-cost fabrication method that is feasible to scale up for industrial air filter manufacturing, whereas electrospinning on a small scale offers rapid response and prototype testing for personalized customizations based on onsite needs [96].

**Figure 4:**
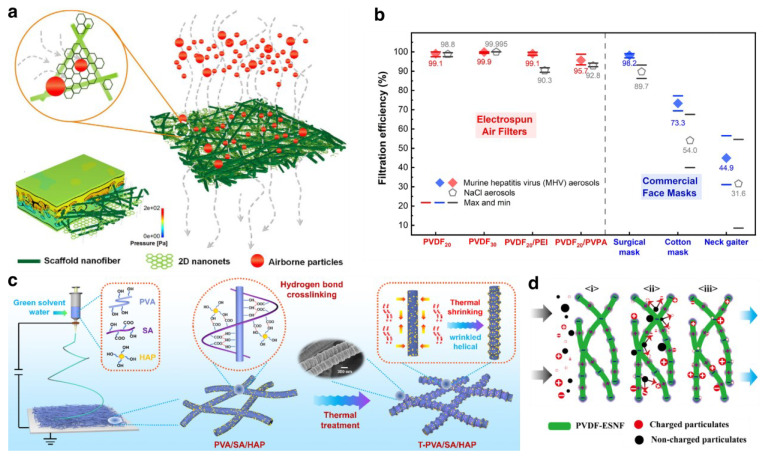
Nanofibrous mat prepared by electrospinning for filtration: (**a**) Filtration works with physical sieving (Adapted and reproduced with permission from Ref. [65]. Copyright 2017, Springer Nature); (**b**) Electrospun air filters have eminently better performance than commercial face masks (Reprinted with permission from Ref. [96]. Copyright 2017, American Chemical Society); (**c**) Secondary structure of helical wrinkled nanofibers introduced by electrospinning (Reproduced with permission from Ref. [66]. Copyright 2022, Elsevier B.V.); (**d**) Self-powered facemask based on electrospun triboelectric nanogenerator (Adapted and reproduced with permission from Ref. [35]. Copyright 2018, American Chemical Society).

Figure 4a shows a PMIA nanofiber filter that efficiently physically sieves particles that are 300–500 nm in diameter, with an ultra-low penetration air filter level of 99.999% [65]. The absolute removal manner is shown in the zoomed-in red circle, and 3D modeling (the lower half) illustrates its robust air permeability. When it comes to applications in face masks, nanofibrous filters prepared by electrospinning clearly have advantages over commercial counterparts prepared by melt-blowing, especially in controlling aerosols. The SARS-CoV-2 virus is shown to have a diameter of less than 1 μm [97], which is tiny enough to slip through surgical masks that hold pore sizes of a couple of micrometers or cloth masks with pore sizes up to tens to hundreds of micrometers. Shen et al. proposed an electrospun nanofiber that can filter out SARS-CoV-2 virus aerogel with ultrafine (~300 nm) nanofibers [96]. The comparison results of aerosol filtration efficiency of electrospun air filters and commercial face masks, shown in Figure 4b, demonstrate a clear superior advantage in filtration efficiency of electrospun air filters over commercial face masks, which are represented with red and blue diamonds, respectively. This electrospun nanofibrous filter shows excellent performance with a 99.9% capture rate of coronavirus aerosols.

The emerging concept of green electrospinning, expounded from the aspects of green degradable materials, solvent-free electrospinning and green solution electrospinning [98] have attracted attentions for the further alliance with eco-friendly, sustainable development and circular economy [99,100]. In this regard, an eco-friendly and biosafe polyvinyl alcohol/sodium alginate/hydroxyapatite (T-PVA/SA/HAP) nanofiber was fabricated through morphology engineering by Deng et al. [66] shown in Figure 4c. Through green electrospinning and thermal treatment, a hierarchical helical nanofiber structure is created to promote filtration performance due to the greatly increased physical interception with particulates. HAP nanoparticles introduced electrostatic adsorption as well, resulting in an overall removal rate of above 99% for particulate matter with a size range of 0.3–2.5 μm. Another example of electrospun nanofibers in respiratory applications is taking advantage of the outstanding dielectric property of PVDF as a triboelectric nanogenerator [35]. Schematic of electrostatic adsorption filtering driven by triboelectric effect (Figure 4d) illustrates that with air flows though the filter carrying charge or neutral particulates, contact electrification of PVDF nanofibers leads to charge polarization and thus captures the charged ones and charges the noncharged particulates. In the end, all charged particulates are absorbed and trapped on the filter membrane. This membrane is then implanted into a face mask where the airflow of the breath and movement driven by respiratory is most concentrated.

## 3. Mask More Than a Filter

Apart from the filtration capability, as the fundamental function of a mask, there are many more roles and applications a mask can fit to fulfil complex application requirements when introduced expanded features and additional functionalities from healthcare, sports tracking, fashion and military applications. Developing new functionalities for wearables, including smart masks, has become a new trend and has the potential to revolutionize the role these devices play in our daily life, reflected by the heated focus on self-cleaning, sensing, actuating and communicating through introducing new materials, structures and nano-coatings [101,102,103]. Many bulky machines and cumbersome wires connect them for communication, which substantially limits patient’s mobility. Not only for the comfort of the person under test, to accurately reflect the medical condition of the object also requires a continuous monitoring session to track signals in real time. Thus, a personalized portable device is needed to seamlessly monitor the physiological signals of the wearer. Tailoring polymeric fibrous filters with multi-functionalities has shown potential in numerous filtration applications. The properties of interest include toxic gas absorption, antibacterial film, anti-corrosive film, etc.

Masks are naturally suitable for serving as a base substrate of wearable electronics for implanting multi-functional modules with individual sensing and/or tracking capabilities. This inherent convenience opens up the possibility and feasibility of applying mask-based smart wearables in various health monitoring applications that respond to multiple input signals [104]. A comparison of smart masks with multimodal sensing capabilities is listed in Table 2.

### 3.1. Intelligent and Green Wearables

The concept of “intelligence” in a material refers to its ability to sense, respond or react to external stimuli or changes in environmental conditions [112,113]. Heated research interests have focused on the synthesis, optimization and application of materials that can respond to their environment or adjust their properties given external stimuli. Increased involvement of such intelligent or smart materials is witnessed in filtration membrane [67,114,115] design. Similarly, the “smartness” of a respirator can be introduced either intrinsically or extrinsically. In the former case, the ability of an air filtration structure to respond and react to circumstantial variation comes from its inherent structure or material. The latter case involves extrinsic supporting modules integrated with the filter to achieve overall multi-functionality.

For smart masks fulfilling the post-pandemic scheme and an intelligent future, there are versatile approaches to design and fabricate respirators that are integrated with smaller, portable or even personalized specific functional modules. At the same time, sustainable materials that are degradable, recyclable or environmentally friendly have caught more attention with increased awareness of developing a circular economy [116,117,118], such as biodegradable and biocompatible materials [98,119,120] especially for skin interface sensors. For example, biocompatible and environmentally friendly options including polydimethylsiloxane (PDMS) or multiscale porous polystyreneblock-poly(ethylene-ran-butylene)-block-polystyrene (SEBS) as substrates, hydrophobic poly(3,4-ethylenedioxythiophene):poly(styrenesulfonate) (PEDOT:PSS) and AgNWs as conductive materials are particularly suitable for wearable electronic applications [121,122,123,124]. Therefore, it is vital to have the principle of sustainable development as a frame within which to derive wearable design.

### 3.2. Macroscopic Physiological Signal Monitoring

With the significant attention on wearable devices, it is well recognized and established that their non-invasive approaches are desirable for physiological signal monitoring [125]. Apart from conventional clinical practice that provides health characterization at considerable cost, wearable systems have great potential thanks to their cost-effective nature and continuous utility [101,126,127]. In fact, a wide range of biosignals generated around the face area are eligible to be captured, observed and analyzed by mask. For general health care monitoring purposes, a set of biosignals on a macroscopic level captured by appropriate sensors attached/integrated in masks serves as an indicator of the health status of the wearer. These captured signals, such as heart rate, respiratory rate and blood oxygen saturation (SpO_2_) level, require subsequent processes to interpret and explain. This makes smart masks incorporated with sensors that react to biosignals or environmental stimuli as wearable healthcare possible [128]. A miniaturized system is preferred due to the relatively limited area for sensor implementation on the mask, while integrating machine learning techniques for the analytical evaluation of complex relationships [129,130,131] is another approach.

Respiration rate, together with blood pressure, body temperature and heart rate, are decisive physiological signals that reflect the object’s health condition. Respiratory detection with rate and depth information is a vital metric and provides diagnostic reference for health status estimation of patients that irregularities in respiratory patterns can be a predictor of potentially grievous clinical events [132,133], or an indicator to characterize illness such as obstructive apneas for Parkinson’s disease [134]. With face masks covering the wearer’s noses, it is undoubtedly convenient to acquire respiratory information. The locations of the integrating sensors on the masks directly determine the sensing functionality. For instance, facial vessels provide information about heart rate, oxygen saturation level, and blood pressure, while skin area covered by a mask when investigated with electrooculography to capture eye blink, electrodermal activity for emotion evaluation, electromyography on facial muscle for emotion or pain detection, or inertial measurement on the head provides motion movement information.

For mask applications, it is often expected that the mask will serve as a strain or tactile sensor that is attached or integrated to itself and, at the same time, conformable and comfortable for the user to wear. In these cases, the user’s activities, such as breathing or coughing, introduce a change in the air pressure within the wearer and mask, resulting in tiny movement or deformation. The movements captured are the stimuli. For example, Li et al. developed a piezoresistive sensor for masks composed of MXene-coated tissue paper that responds to pressure changes induced by human movements [135]. Tissue paper was immersed in the MXene solution, followed by vacuum drying to obtain Mxene-coated tissue paper. Uniformly coated MXene nanosheets on the flexible substrate are compressed and move closer when pressure is applied, thus creating more conductive pathways and expanded contact areas with interdigitated electrodes, resulting in resistance changes. In this way, the input mechanical deformation is converted to electro-signals that are quantified. They further configured an array layout of multiple piezoresistive sensors, where the spatial distribution of pressure applied to reflect respiratory conditions could be remotely monitored via wireless communication.

**Figure 5:**
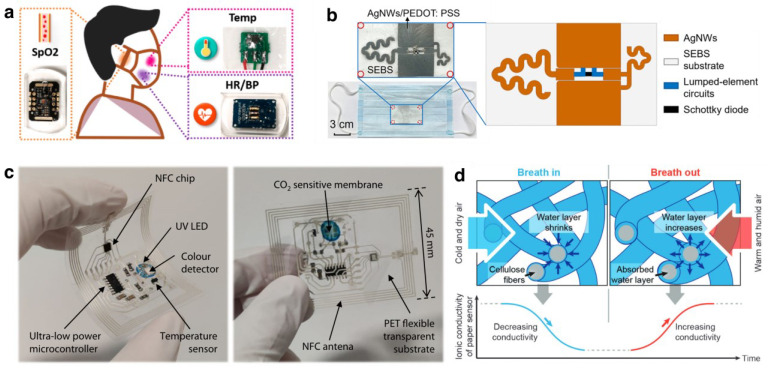
Mask detects macroscopic physiological signals: (**a**) A smart mask integrated with a sensor system that can remotely monitor variables like respiratory rate, heart rate, skin temperature and blood oxygen saturation (Reproduced with permission from Ref. [105]. Copyright 2020, American Chemical Society); (**b**) A self-powered mask with a sensor that detects coughs and wearing by mask deformation (Adapted and reproduced with permission from Ref. [136]. Copyright 2022, American Chemical Society); (**c**) A smart mask with CO_2_ sensitive membrane that wirelessly detects CO_2_ level around the wearer (Adapted and reproduced with permission from Ref. [137]. Copyright 2022, Springer Nature); (**d**) A cellulose paper-based mask that functions as a moisture sensor (Reprinted with permission from Ref. [138]. Copyright 2016, Wiley-VCH GmbH, Weinheim).

Respiratory detection through face mask attached sensors includes sensors working with mechanisms other than strain. Pan et al. proposed a concept of “lab-on-mask” design that integrates a noncontact multiplexed sensor system on a mask for respiratory monitoring [105] shown in Figure 5a.

They embedded multiple sensors for heart rate, SpO_2_, body temperature, and blood pressure monitoring on PDMS as the substrate before attaching on a surgical mask, due to PDMS, which has a similar Young’s modulus to human skin and overcomes the difficulties in commercial printed circuit boards being unconformable and incompatible with human skin [139]. Different sensors collected data from the face surface, and the recorded data were then sent to the receiving and processing terminal. Specifically, through a green light-emitting diode (LED), the photoplethysmography sensor detects and converts light signals reflected from blood vessels beneath the epidermis to electrical signals and realizes the detection of heart rate. A similar mechanism applies for SpO_2_ detection via infrared LED. Finally, after separate preamplifiers, the physiological data collected from each module were sent to the microprogrammed control unit (MCU) for processing and transferred to the receiving terminal via Bluetooth. Another example of wireless monitoring of respiratory behaviors by smart mask proposed by Ye et al. [136] involves a radio frequency harmonic transponder that doesn’t require a battery to power. Specifically, PEDOT:PSS coated silver nanowire on a porous substrate composed of the transponder and performed a tree-like geometry that maximized the antenna configuration (Figure 5b). The device was pasted to the inner layer of the surgical mask, where it responds to abnormal conditions, such as coughing or improperly worn mask. This passive wireless sensing ability is achieved thanks to its tree-shaped antenna design that resonates with a fundamental frequency of 1.6 GHz, with a frequency multiplier, curve-shaped antenna, which resonates with a second-harmonic frequency of 3.2 GHz. As such, measuring the reflection coefficients between these two provides information of antenna distance becomes above 10 mm such that when receiving antenna detects pulsed second harmonic, it can be implied that a coughing behavior is presented, or the mask is not in its properly worn condition. In addition, the device can be torn apart from the mask from assigned stick points after use, making it possible to recycle and reuse the material.

On the other hand, face masks can sense and reflect environmental information from air quality, and environmental temperature to humidity level surrounds the user as well. For instance, Escobedo et al. proposed a wireless gaseous CO_2_ level detector deposited on a flexible tag that sticks to the inner side of a standard FFP2 facemask [137]. The developed CO_2_ sensor works with the mechanism that the acid–base indicator inflects the luminescence level of an inorganic phosphor La_2_O_2_S:Eu, and with UV LED and color sensor, the acidity of gaseous CO_2_ is indicated. The sensing module together with UV LED, color detector, temperature sensor and supporting circuits were printed on a flexible polyethylene terephthalate (PET) substrate, shown in Figure 5c. Apart from CO_2_ level, face masks with temperature or humidity sensing capabilities are developed by researchers as well. Firat et al. developed a respiration sensor based on cellulose paper, with printed graphite as electrodes and attached in a surgical mask, in response to humidity level change [138]. Figure 5d shows it’s moisture sensing mechanism: when the wearer exhales, the humidified moist air goes out with RH 100% passing through the cellulose fibers and changes their ionic conductivity, which is found to be proportional to the amount of water on the fiber surface. During inhale, the moisture adsorbed by cellulose fiber reduces because the surrounding atmosphere always holds a lower RH than exhaled air. In this way, the hygroscopic cellulose paper-based humidity sensor measures the respiratory pattern of the wearer by reflecting cyclic inhale/exhale behaviors to electrical signal, and it serves as a convenient, low-cost, and environmentally friendly wearable for personal health care.

### 3.3. Disease Diagnosis and Monitoring

**Figure 6:**
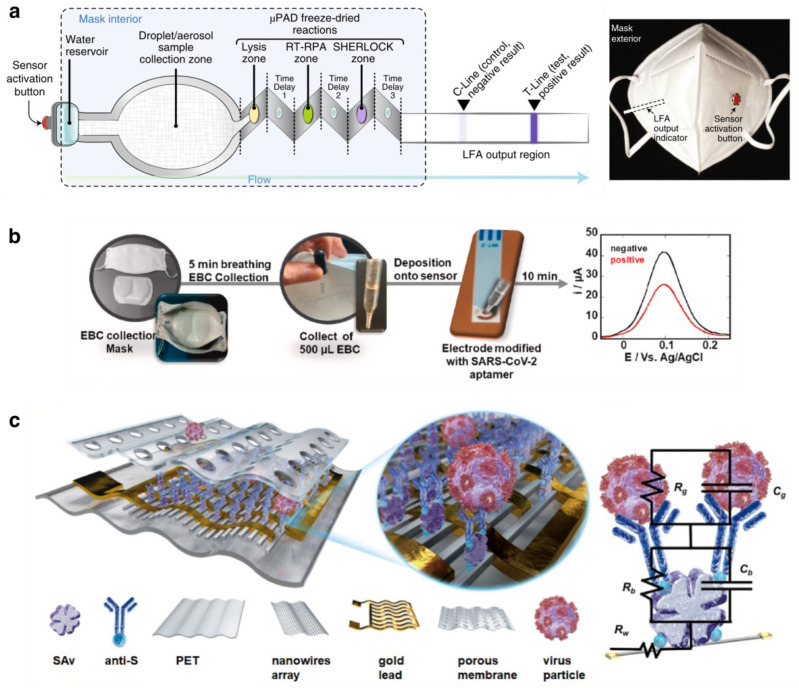
Mask-based COVID-19 diagnosis: (**a**) A face-mask-integrated sensor for SARS-CoV-2 detection in exhaled aerosols (Adapted and reproduced with permission from Ref. [140]. Copyright 2021, Springer Nature); (**b**) A mask-based diagnostic platform for point-of-care screening of COVID-19 (Reprinted with permission from Ref. [141]. Copyright 2021, Elsevier B.V.); (**c**) An intelligent face mask integrated with a high-density conductive nanowire array for directly exhaled coronavirus aerosol screening (Reprinted with permission from Ref. [142]. Copyright 2021, Elsevier B.V.).

Bio-integrated wearable devices offer economic characterization, portable health care analysis, and continuous monitoring are suited to fill in gaps in conventional medical practice [125]. For example, mask-based wearables for glucose monitoring [32] where metal nanowires with enzyme modified electrodes for H_2_O_2_ and glucose sensing, and other types of nanowire modified electrodes for different types of sensors for chemical sensors and biosensors [79]. Physiological signals captured by smart masks offer a convenient approach for disease diagnosis and monitoring, and when equipped with biosensors, they are specifically suitable for respiratory diseases such as COVID-19 since the evidence of abnormal breathing patterns reflects directly on masks. Figure 6 shows the mask-based COVID-19 diagnosis designs. In Figure 6a, Nguyen et al. reported a biomolecule detection embedded on a facemask with synthetic biology sensors that provides SARS-CoV-2-sensing though freeze-dried reactions on a microfluidic paper-based analytical device, which prototype rapidly and feasible to regulate optimization of the multi-step reactions [140]. When the virus accumulates in the mask with the patients’ respiratory behavior and flows through the puncture of the water blister reservoir, viral particles are transported from the collection sample pad to the microfluidic paper-based analytical device downstream due to capillary action. The three reaction zones are separated by PVA to ensure sufficient time delay for incubation. After lyophilized lysis reagents, isothermal amplification targeting for a nonoverlapping region of the SARS-CoV-2 S gene and amplified dsDNA amplicon detection, a lateral flow assay strip visualized the results. Notably, this mask-based biosensor doesn’t require laboratory equipment or specialists like other nucleic acid testing demands; it operates on an autonomous base and offers safe, efficient testing without further concerns.

Daniels et al. used exhaled breath condensate instead of exhaled breath as the specimen for COVID-19 diagnostic [141]. This mask-based diagnostic platform collects the exhaled breath condensate, detect and quantify SARS-CoV-2 virus via an electrochemical biosensor with a modular architecture, making it a fast and cost-effective alternative for reverse-transcription polymerase chain reaction (RT-PCR) based on saliva swab test (Figure 6b). However, to effectively secure the exhaled breath condensate collection, it is necessary for the mask to be cooled in the freezer for 30 min prior to a 5 min collecting period, which sacrifices the convenience of the testing process. Exhaled breath condensate is then deposited onto the sensing electrode through a designed line inside the mask, which is modified with an aptamer that responds to the SARS-CoV-2 spike protein.

Xue et al. also reported a direct exhaled coronavirus aerosol screening face mask using anti-spike proteins of SARS-CoV-2 [142]. The sensor consists of three layers: a droplet collecting protective outer layer of polycarbonate membrane, sensing bio-functional middle layer, and a PET supportive substrate to adhere to on the mask (Figure 6c). As shown, the equivalent resistance and capacitance of surface binding antibody are denoted as R_b_ and C_b_, respectively, whereas R_g_ and C_g_ represents equivalent resistance and capacitance of surface binding antigen, and R_w_ represents the equivalent resistance of PEDOT:PSS nanowires doped with the biotin groups. With this impedance circuit model, spike protein detection is simulated by impedance measurement: once viral particles are captured, the nanowire within two adjacent electrodes exhibits as a resistor R_w_ with R_g_ parallel to C_g_, resulting in an equivalent circuit in series. This means that with a viral aerosol concentration increase, indicating more antigens to be captured, the overall impedance of antigens increased, so as the whole system measurement. Face mask integrated biosensors offer an efficient and non-invasive detection approach, making them promising in early stage disease detection, such as exhaled M tuberculosis for diagnosis of Tuberculosis [143].

## 4. Next-Generation Mask Design

Smart filters that are applicable to wearable PPE emphasize their portability. One must consider the trade-off between mask filtration performance and the comfort for users to wear since the filtration efficiency is almost inversely proportional to respiratory resistance [25]. Thus, improving the filtration efficiency of the filtration material while limiting the increase in pressure drop that contributes to the respirations of the face mask can be one of the future research directions. In addition, ergonomic design for usability [128] is another consideration that masks with suitable shapes function effectively for a longer time period, while non-certified masks or individual errors could lead to ineffective mask fitting [7,144]. As mentioned in Section 2.2, the filtration efficiency of a mask is also determined by its leakage potential and how well the mask fits the wearer. For example, studies have shown that cone-shaped masks exhibit a smaller peripheral while maintaining a consistently well-tensioned fitting over flat-shaped surgical masks [145]. On the other hand, current surgical or N95 masks with nano-scale-sized functional materials are reported to cause overall discomfort, lower skin temperature and significantly lower hear rates [146], meaning smart masks with good air and water vapor permeability are in need. Besides, reusability, recyclability and end-of-life processing of a mask is necessary since masks can be a source of contamination if not disposed of or replaced in a timely manner [90,147].

Face masks as wearables with integrated multifunctional sensors that detect human body physiological signals and surrounding environmental status have broadened the practical applications of their conventional function as air filters. Earlier research development of sensors integrated on masks was considered to functionalize the available masks in the market, while considering the material and structural design of the mask itself brings more possibilities and promising realizations in daily health monitoring, sports training, ambulatory settings, PPE in special working environments, etc. The rise of these intelligent devices with features that are compact, flexible, self-powered, or sustainable pushes forward innovative advanced biosensor wearable development [148,149,150]. It can be envisioned that next-generation masks are smart to sense and react to different stimuli or environmental situations, composed of biocompatible and sustainable materials, and scalable to manufacture and append functionalities with customizable demands.

## Figures and Tables

**Table 2:** Smart masks with multimodal sensing capabilities.

Multimodal Sensing	Sensing Mechanism	Characteristics	Ref.
Heart rate, SpO_2_	Photoplethysmography sensor	Long-time and real-time remote monitoring of vital signs	[105]
Skin temperature	Thermistor sensor		[105]
Multiphase respiratory activities	Piezo-impedance property of CNT/PDMS	A wide bandwidth dynamic pressure range with data processing and classification by machine learning	[106]
Cardiorespiratory monitoring of ECG ^1^, LVET ^2^, PEP ^3^	ECG via a wireless, multimodal stethoscope patch and validates LVET and PEP estimation from respiratory flow through a facial mask	Good performances with patient in the supine, lateral, and prone position	[107]
Cough events	Audio and accelerometer sensors from multiple devices	Cross-correlation based adaptive time synchronization algorithm to ensure accurate time synchronization during concise event of cough	[108]
Respiration monitoring and OSAS ^4^ diagnose	Capacitance and resistance change of cellulose-based hydrogel	Capacitance of hydrogel sensor changes with mechanical variation and resistance responds to thermal stimulus	[109]
Dampness and respiration rate monitoring	Printed interdigitated electrode patterns to sense resistive and capacitive change by exhaled humidity	Identify breathing patterns and evaluate infection signs	[110]
Facial and head physiological signals	3 sensor modalities to measure facial muscle movements and motions	ML assisted analyzation of wearer’s context and affective state	[111]

^1^ ECG: Einthoven electrocardiogram. ^2^ LVET: Left ventricular ejection time. ^3^ PEP: Pre-ejection period. ^4^ OSAS: Obstructive sleep apnea syndrome.
